# Group Model Building on causes and interventions for falls in Singapore: insights from a systems thinking approach

**DOI:** 10.1186/s12877-023-04294-2

**Published:** 2023-09-22

**Authors:** Wei Xuan Lai, Angelique Wei-Ming Chan, David Bruce Matchar, John Pastor Ansah, Christopher Tsung Chien Lien, Noor Hafizah Ismail, Chek Hooi Wong, Tianma Xu, Vanda Wen Teng Ho, Pey June Tan, June May Ling Lee, Rita Siew Choo Sim, Normala Manap

**Affiliations:** 1 Duke-NUS Medical School, Programme in Health Services and Systems Research, 8 College Road, Singapore, 169857 Singapore; 2 Centre for Ageing Research and Education, Duke-NUS Medical School, Singapore, Singapore; 3 Department of Medicine, Duke University, Durham, NC USA; 4 Singapore-ETH Centre, Future Health Technologies Programme, CREATE Tower, Singapore, Singapore; 5 Center for Community Health Integration, Case Western Reserve University, Cleveland, OH USA; 6 Department of Geriatric Medicine, Changi General Hospital, Singapore, Singapore; 7 Department of Continuing and Community Care, Tan Tock Seng Hospital, Singapore, Singapore; 8 Institute of Geriatrics and Active Ageing, Tan Tock Seng Hospital, Singapore, Singapore; 9 Geriatric Education and Research Institute, Singapore, Singapore; 10 Health & Social Sciences Cluster, Singapore Institute of Technology, Singapore, Singapore; 11 Department of Medicine, National University Hospital, Singapore, Singapore; 12 Ageing and Age-Associated Disorders Research Group, Health and Translational Medicine Cluster, University of Malaya, Kuala Lumpur, Malaysia

**Keywords:** Group model building, Systems thinking, Falls prevention, Older adults

## Abstract

**Background:**

Falls in older adults are the result of a complex web of interacting causes, that further results in other physical, emotional, and psychological sequelae. A conceptual framework that represents the reciprocal dynamics of these causal factors can enable clinicians, researchers, and policymakers to clarify goals in falls intervention in older adults.

**Methods:**

A Group Model Building (GMB) exercise was conducted with researchers and clinicians from academic units and public healthcare institutes in Singapore. The aim of the exercise was to produce a shared visual representation of the causal structure for falls and engage in discussions on how current and future falls intervention programmes can address falls in the older adults, especially in the Asian context. It was conducted in four steps: 1) Outlining and prioritising desirable patient outcomes, 2) Conceptual model building, 3) Identifying key intervention elements of effective falls intervention programmes, 4) Mapping of interventions to outcomes. This causal loop diagram (CLD) was then used to generate insights into the current understanding of falls causal relationships, current efforts in falls intervention in Singapore, and used to identify gaps in falls research that could be further advanced in future intervention studies.

**Results:**

Four patient outcomes were identified by the group as key in falls intervention: 1) Falls, 2) Injurious falls, 3) Fear of falling, and 4) Restricted mobility and life space. A CLD of the reciprocal relationships between risk factors and these outcomes are represented in four sub-models: 1) Fear of falling, 2) Injuries associated with falls, 3) Caregiver overprotectiveness, 4) Post-traumatic stress disorder and psychological resilience. Through this GMB exercise, the group gained the following insights: (1) Psychological sequelae of falls is an important falls intervention outcome. (2) The effects of family overprotectiveness, psychological resilience, and PTSD in exacerbating the consequences of falls are not well understood. (3) There is a need to develop multi-component falls interventions to address the multitude of falls and falls related sequelae.

**Conclusion:**

This work illustrates the potential of GMB to promote shared understanding of complex healthcare problems and to provide a roadmap for the development of more effective preventive actions.

**Supplementary Information:**

The online version contains supplementary material available at 10.1186/s12877-023-04294-2.

## Introduction

Falls in older adults are common and serious. Worldwide, approximately one in three older adults above the age of 60 experience at least one fall every year [1–3] and, those who fall are more likely to fall again within the next year [4, 5]. Falls are the leading cause of injury in older adults, accounting for 65%-90% of trauma cases seen in emergency departments [6, 7]; about one third of such cases are associated with major physical sequelae, such as head and face injuries and upper limb fractures [7, 8]. Beyond the physical consequences, individuals who fall often experience emotional distress [9, 10] and tend to restrict their mobility [11, 12]. With its rapidly ageing population, falls in older adults are becoming an increasingly urgent public health problem in Singapore. About 13.4%- 17.2% of older adults living in the community experience at least one fall every year, with a third of those having recurrent falls within the same year [13, 14].

Numerous studies have sought to identify the risk factors for falls in order to adopt strategies that will alleviate the risks for high-risk groups [10, 15–17]. Falls are caused by multiple factors that vary between people, hence, a tailored approach to minimise risk and prevent falls in specific groups of people have been previously adopted [18–20]. Although several efforts have been made to identify and address the multiple risk factors for falls, a systems perspective on how these factors interact with each other to influence the likelihood of falls is lacking. Currently, there are no unifying frameworks that examine how individual, family, and environmental factors interact to cause falls and its related sequelae. Furthermore, there have been few efforts to pull together a broad perspective on the impact of falls and its related sequelae on fallers, their families, and community efforts to prevent falls. Therefore, falls in older adults have become a clinical and public health concern that must be addressed by (1) understanding and alleviating the root causes of falls, (2) recognizing the physical and emotional sequalae of falls, and (3) gaining a systems perspective on how the factors can influence the success of strategies adopted to minimise the negative effects of falls.

Although falls in older adults is a global public health problem, prevention of falls is particularly challenging in Asian countries. In a review of falls prevention research conducted in Asia, Hill et al*.* found that only 11 of 30 interventions demonstrated a significant reduction in falls. Furthermore, only exercise interventions were found to be effective in reducing falls, with multi-component interventions showing poorer success. Translating the success of falls intervention approaches from western countries into the Asian context remains difficult due to underlying differences in several aspects, including (1) the role of family, (2) lifestyle, (3) health services and systems, (4) changes in behaviour due to anxiety from a previous fall, (5) adoption of falls prevention strategies [21].

With one of the most rapidly ageing populations in the world, Singapore has made falls prevention a major public health objective [22]. Multiple research units and public healthcare institutions have developed intervention programmes to tackle modifiable risk factors for falls and prevent recurrent falls. These include the Steps to Avoid Falls in the Elderly (SAFE) programme, a randomised controlled trial to assess the effectiveness of progressive physical therapy [19], as well as a modified version of the Stepping On Programme [23, 24]. Amid ongoing research, several operational efforts to reduce falls have been introduced at the national and regional levels.

In this context, a meeting was convened on the 26^th^ of September 2019 by stakeholders involved in falls burden reduction to develop a strategic approach that will integrate research findings with practical approaches to effectively prevent falls in the community. Specifically, the group applied the method of Group Model Building (GMB) to develop a shared hypothesis about the causal structure for falls and its related sequelae, certain aspects of which could be leveraged for designing interventions that may prevent future falls. In addition, the group identified areas where additional data from research is needed to inform clinical and public policymakers, in Singapore as well as globally. In this report, we describe the GMB exercise and the insights that emerged from its application. Our goal is to introduce GMB as a strategy for promoting shared understanding of complex problems, as well as to highlight the significance of GMB in developing a common roadmap for falls prevention strategies in order to improve public health outcomes.

## Methods

### Participants

The GMB exercise was co-organised by two academic units in Singapore—the Geriatric Education and Research Institute (GERI) and the Centre for Ageing Research and Education (CARE). In the exercise, thirteen falls researchers with experience conducting falls research in the three different healthcare clusters in Singapore participated to lend their expertise on falls intervention. In this stakeholder group, four of the researchers are practicing geriatricians, and one was trained as an occupational therapist. The stakeholders are well represented across five public healthcare institutions and two health services academic units in Singapore. The stakeholders participating in the GMB exercise have each conducted a formalised community-based falls intervention program in Singapore as part of their clinical, or research activities. These stakeholders provided their insights in order to build the framework for the causal structure of falls and understanding falls intervention in Singapore.

### Group model building exercise

Group Model Building (GMB) denotes a series of exercises that are performed by a group of people with diverse perspectives and objectives, to solve a complex problem [25]. The result of the exercise is a visual representation of the causal structure of the problem, including important outcomes, factors leading to those outcomes, and the intended and unintended consequences that may arise from planned efforts.

**Fig. 1:**
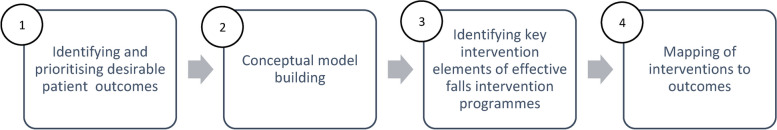
Workflow of group model building exercise for conceptualising the causative structure of falls, mapping current falls interventions and identifying gaps in interventions

The exercise follows a general script (Appendix) involving constant group participation, engaging each individual in sequence, and building on each other’s inputs through a series of steps that maintains momentum in the group. In this case, the script followed the workflow illustrated in Fig. 1, and is comprised of four steps: 1) Outlining and prioritising desirable patient outcomes, 2) Conceptual model building, 3) Identifying key intervention elements of effective falls intervention programmes, 4) Mapping of interventions to outcomes. Though customized to this GMB exercise evaluating falls intervention research in Singapore, the script generally follows the steps and sequences in GMB exercises evaluating healthcare services [26, 27].

Briefly, the four steps in this GMB exercise were conducted as follows:

- (1) Outlining and prioritising desirable patient outcomes. At the start of the GMB exercise, stakeholders were asked to identify key outcomes in falls prevention implementation research. They first responded with a number of hopes and fears related to the exercise, ranging from its usefulness to the potential impact that the group can achieve by developing a shared understanding of falls research conducted in Singapore. Then, they reflected on the outcomes of falls in older adults in Singapore and considered whether each outcome listed was important in and of itself, i.e., the outcome was not only important solely because it caused another outcome. Following this, the group identified a set of desirable patient outcomes that would be significant enough to justify investing considerable effort and resources into developing falls prevention intervention programs.
  The group was then asked to prioritise key outcomes for implementation research in falls. The outcomes were ranked based on the results of a poll taken of the stakeholders present in the GMB exercise.
- (2) Conceptual model building. The goal of conceptual model building in GMB is to represent the problem in a way that is agreed upon by group members as reflecting known or hypothesized cause-effect relationships – a form of “boundary object” [28]. In this case, the value of working with a translational group was that they represented diverse perspectives – medical, social, and psychological – about the complex problem of falls in older adults. It was challenging to incorporate these diverse perspectives in one conceptual model comprising several domains, which can interact in direct and reciprocal ways.
  The most common visual representation used in GBM is a Causal Loop Diagram (CLD), a qualitative system thinking structure that is based on a simple visual vocabulary and construction steps (Table 1). First, key outcomes and causes are identified and placed on the diagram. Then, arrows are used to represent the cause-and-effect relationship, with the direction of the arrow indicating the direction of the relationship, and a ‘ ± ’ symbol indicative of the polarity of the relationship; a ‘ + ’ sign indicates a positive effect, meaning that an increase in the cause variable, all other variables being equal, will result in an increase in the effect variable. Alternately, a ‘-’ sign indicates a negative effect, meaning that an increase in the cause variable, all other things being equal, will result in a decrease in the effect variable. Lastly, reciprocal relationships between variables in the CLD that form loops are identified. Causal loops that tend to promote progressive increase of variables contained within the loop are termed “reinforcing” while causal loops that tend to counter an increase of variables within the loop are termed “balancing”. A summary of the symbols and notations commonly used in CLDs can be found in the Appendix.
  In our study, the CLD built by the group was sufficient to achieve the primary objective of the GBM exercise. This CLD was then used to facilitate the discussion in the subsequent steps of the GMB exercise.
- (3) Identifying key intervention elements of effective falls intervention programmes. After building the CLD, the group highlighted the key elements of effective falls intervention strategies used in programmes that they had previously conducted.
- (4) Mapping of interventions to outcomes. They then discussed how current intervention programmes targeted specific variables in the CLD that they built. This allowed the group to identify ways in which the current intervention programme addressed the underlying causes of falls and its associated physical, emotional, and psychological sequelae.

**Table 1:** Definitions of symbols used in causal loop diagrams

Symbol	Definition
	A “ + ” sign denotes that an increase in variable “A” leads to an increase in variable “B”, or a decrease in variable “A” leads to a decrease in variable “B”, all things being equal. That is, variable “A” has a positive relationship with variable “B”
	A “ + ” sign denotes that an increase in variable “A” leads to a decrease in variable “B”, or a decrease in variable “A” leads to an increase in variable “B”, all things being equal. That is, variable “A” has a negative relationship with variable “B”
	The “R” denotes that the feedback loop is reinforcing, whereby an increase in “A” causes an increase in “B”, leading to a further increase in “A”. A decrease in “A” can also cause a decrease in “B” leading to further decrease in “A”. The feedback loop results in subsequent exponential change—either growth or decay
	The “B” denotes that the feedback loop is balancing, whereby an in “A” causes an increase in “B”, leading to a consequent decrease in “A”. Also, a decrease in “A” can cause a decrease in “B”, leading to a consequent increase in “A”. A balancing feedback loop will reach its limit over time and seeks an equilibrium
	“R1” denotes reinforcing loop 1, a unique identifier given to each reinforcing feedback loop identified. A short description of the feedback loop is included for ease of reference. A table summarising the feedback loops identified traces the causal pathway of each loop. This is done by following the order of the variables described in each feedback loop
	“B1” denotes balancing loop 1, a unique identifier given to each reinforcing feedback loop identified. A short description of the feedback loop is included for ease of reference. A table summarising the feedback loops identified traces the causal pathway of each loop. This is done by following the order of the variables described in each feedback loop

### Data analysis

For the current presentation of the CLD, the authors of this paper transferred the diagram to suitable software (Vensim® by Ventana Systems, Inc). The CLD was evaluated using the set of rules under the Categories of Legitimate Reservation (CLR) [29, 30]. This was done to assure that entities in the CLD are defined explicitly and that the hypothesized causal relationships can be evaluated empirically. The final diagram was reviewed by the group members, and they concurred that the essence of their discussion and the original CLD was captured in the revised version.

## Results

### Participants in group model building session

We invited clinicians and falls researchers across the three healthcare clusters in Singapore, and five different health services research units to give their expert opinion on current falls research in Singapore. The group included four geriatricians, an occupational therapist and four falls researchers. Between the stakeholders, the group conducted three independent community-based falls intervention programs in Singapore [19, 31, 32].

### Priority patient outcomes

Four key patient outcomes were identified as significant in falls implementation research: (1) Falls, (2) Injurious falls, (3) Fear of falling, and (4) Restricted mobility and life space. The study group noted that these outcomes are interrelated. For example, falls can lead to injury. Injurious falls can lead to fear of falling. Fear of falling can lead to restrictions in mobility and life space. Finally, restrictions in mobility and life space can result in fewer falls. These relationships are described in detail below.

### Conceptual model of falls

A causal loop diagram representing the conceptual model of falls was created with four different sub-components: 1) Fear of falling, 2) Injuries associated with falls, 3) Caregiver overprotectiveness, 4) PTSD and psychological resilience.

### Fear of falling

**Fig. 2:**
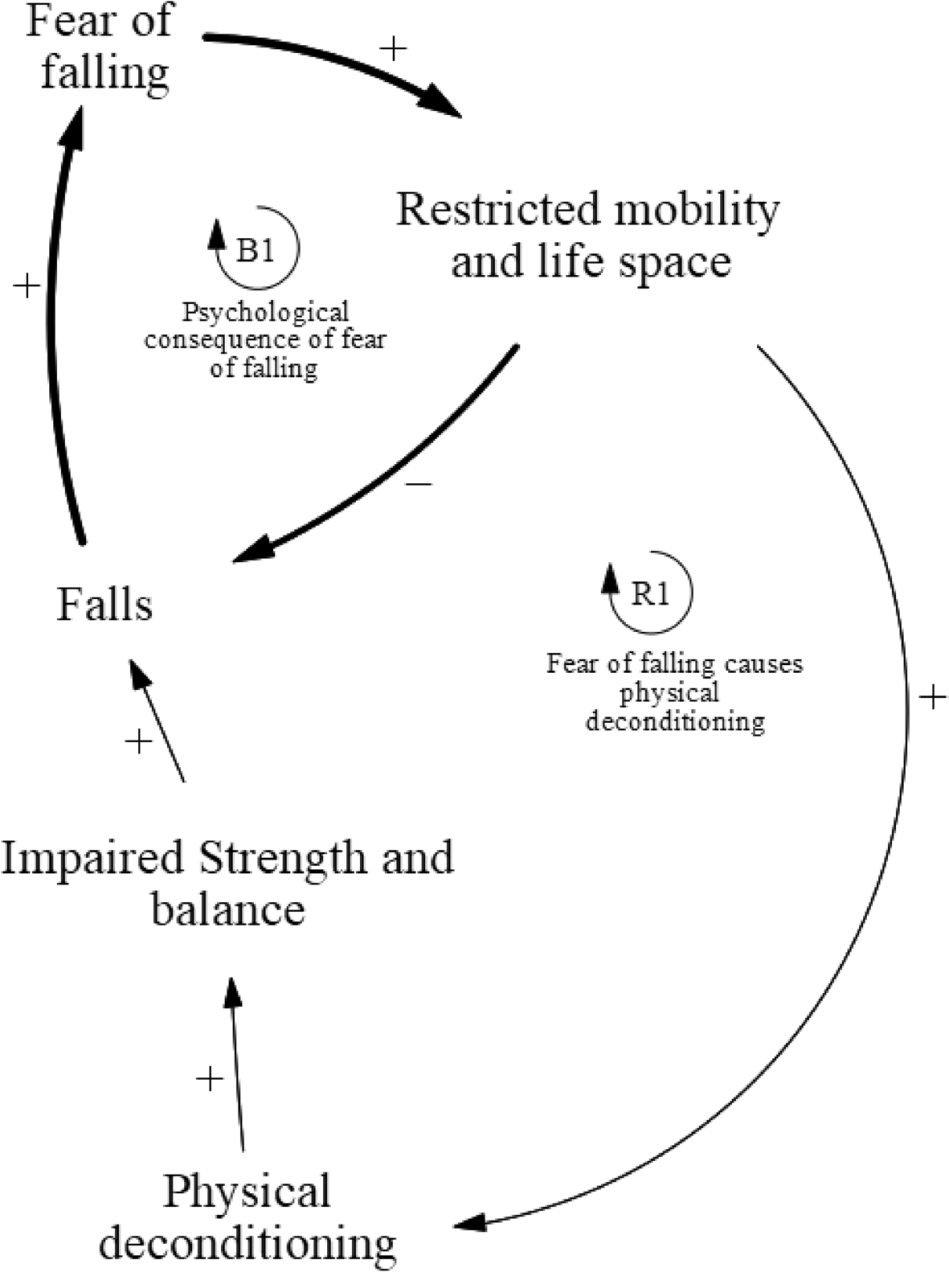
Causal pathways of fear of falling

The fear of falling sub-component of the causal loop diagram (Fig. 2) consists of two feedback loops: balancing loop B1 and reinforcing loop R1 (Table 2). This sub-component describes the emotional and psychological consequences of falling on an individual and how fear of falling plays a role as an important risk factor for falls. Balancing loop B1 stipulates that fallers may develop fear due to the negative emotional and psychological effects of falls, and as a result restrict their mobility and subsequently life space. This may decrease the likelihood of them falling again in the near future. However, reinforcing loop R1 describes how restriction in mobility and life space may result in increased physical deconditioning, which could impair muscle strength and balance in fallers in the long run and lead to more falls in the future.

**Table 2:** Fear of falling feedback loops

Loop	Variables
B1	Falls ➔ Fear of falling ➔ Restricted mobility and life space ➔ Falls
R1	Falls ➔ Fear of falling ➔ Restricted mobility and life space ➔ Physical deconditioning ➔ Impaired strength and balance ➔ Falls

### Injuries associated with falls

**Fig. 3:**
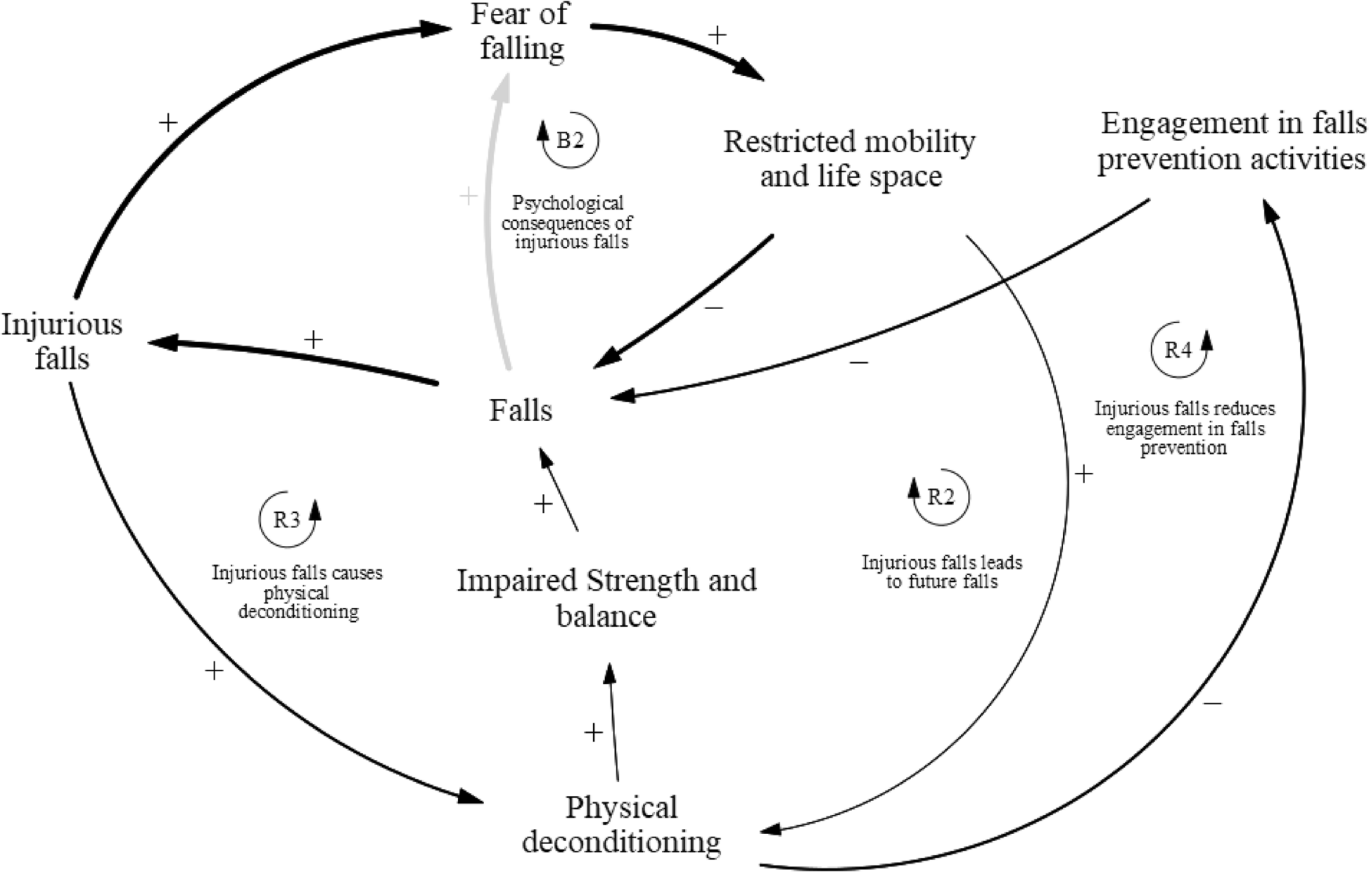
Causal pathways of injuries associated with falls

The injurious falls sub-component of the causal loop diagram (Fig. 3) consists of four feedback loops: one balancing loop B1 and three reinforcing loops R2, R3, and R4 (Table 3). This sub-component describes how injurious falls lead to further physical and psychological sequelae. Balancing loop B2 describes the psychological consequences of injurious falls. Falls that result in physical injuries may induce a fear of falling in fallers. As a result, these fallers might subsequently restrict their mobility and life space. This will decrease the likelihood of a fall in the near future. Reinforcing loop R2 describes how injurious falls predispose fallers to future falls. The trauma from injurious falls may cause the faller to avoid physical activity due to a fear of falling, which could lead to further physical deconditioning and predisposition to future falls. Reinforcing loop R3 describes how injurious falls cause physical deconditioning. Injuries resulting from a fall may necessitate prolonged bed rest or avoidance of physical activity to nurse the injury. This physical deconditioning leads to impaired strength and balance in fallers and therefore result in more falls in the long run. Reinforcing loop R4 describes how injurious falls reduce engagement in falls prevention activities. Fallers who experience increased physical deconditioning after an injurious fall have a limited ability to engage in falls prevention activities. This may be due to physical limitations posed by their previous injuries, preventing them from performing rehabilitative activities, or due to the lack of time and motivation to participate, as they may be still recovering from their injury. Altogether, decreased engagement in falls prevention activities may result in more falls in the future.

**Table 3:** Injurious falls feedback loops

Loop	Variables
B2	Falls ➔ Injurious falls ➔ Fear of falling ➔ Restricted mobility and life space ➔ Falls
R2	Falls ➔ Injurious falls ➔ Fear of falling ➔ Physical deconditioning ➔ Impaired strength and balance ➔ Falls
R3	Falls ➔ Injurious falls ➔ Physical deconditioning ➔ Impaired strength and balance ➔ Falls
R4	Falls ➔ Injurious falls ➔ Physical deconditioning ➔ Engagement in falls prevention activities ➔ Falls

### Caregiver overprotectiveness

**Fig. 4:**
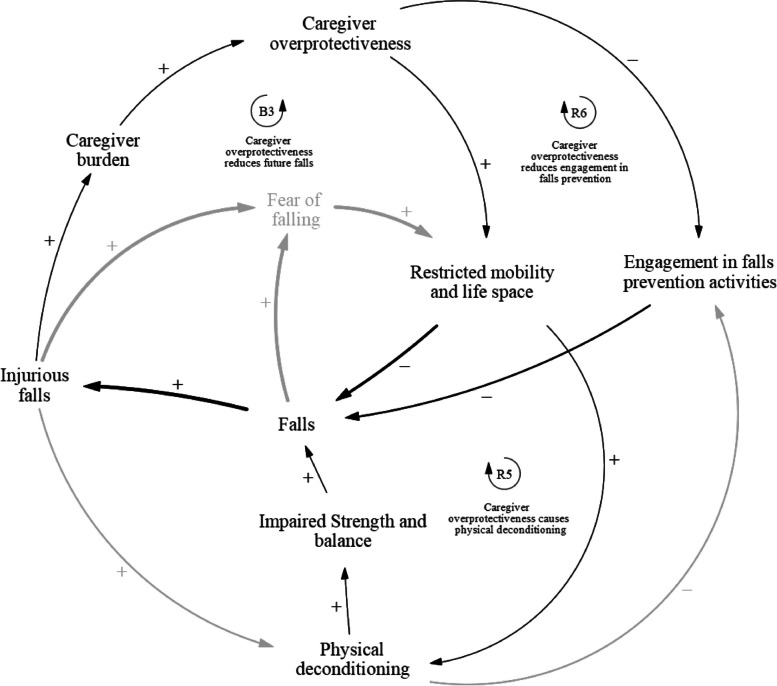
Causal pathways that involve caregiver overprotectiveness

The caregiver overprotectiveness sub-component of the causal loop diagram (Fig. 4) consists of three feedback loops: one balancing loop B3, and two reinforcing loops R5 and R6 (Table 4). This sub-component shows how overprotectiveness shown by caregivers can predispose fallers to future falls and reduce the effectiveness of falls prevention activities. Balancing loop B3 describes how caregiver overprotectiveness reduces future falls. Falls that result in injuries may limit physical activity in fallers, thereby increasing the burden on caregivers. Caregivers may also experience financial burden due to the loss of income from the faller who may not be able to continue working either as a result of poor physical health after a fall, or due to the fear of falling again while working. Caregivers may also choose to stop working to meet the increased care needs of a faller who requires close supervision. This may further increase the financial burden on the caregiver as household income decreases. Overall, the increased burden might cause the caregivers to become more protective of the faller, by restricting the faller's mobility and life space. They may do so by not bringing the faller outdoors and limiting their movement inside homes. In Singapore, as in many Southeast Asian countries, foreign domestic workers are routinely hired as the primary caregiver for older adults with health issues in the household. These workers are equally likely, if not more, than family members to be overprotective of their charges to safeguard their livelihoods. This overprotectiveness results in less falls in the short run as the fallers are under close supervision by family members or workers. Reinforcing loop R4 describes how caregiver overprotectiveness may cause physical deconditioning in the long run. Falls cause the caregiver to become overprotective, which will subsequently restrict the mobility and life space of the faller. However, the restrictions result in increased physical deconditioning of the faller, subsequently impairing their strength and balance and predisposing them to more falls in the long run. Reinforcing loop R5 describes how caregiver overprotectiveness, resulting from increased burden and stress after a fall, may reduce faller’s engagement in falls prevention activities. Caregivers may restrict faller’s participation in falls prevention activities, such as not letting them participate in intense physical exercises, due to concerns about their physical condition. Furthermore, the financial burden from healthcare services and falls prevention programs may prevent caregivers from supporting these falls prevention activities. This may limit the effects of physical exercise interventions and result in less effective falls prevention programmes, thereby increasing the likelihood of future falls.

**Table 4:** Caregiver overprotectiveness feedback loops

Loop	Variables
B3	Fall ➔ Injurious falls ➔ Caregiver burden ➔ Caregiver overprotectiveness ➔ Restricted mobility and life space ➔ Falls
R5	Falls ➔ Injurious Falls ➔ Caregiver burden ➔ Caregiver overprotectiveness ➔ Restricted mobility and life space ➔ Physical deconditioning ➔ Impaired strength and balance ➔ Falls
R6	Falls ➔ Injurious Falls ➔ Caregiver burden ➔ Caregiver overprotectiveness ➔ Engagement in falls prevention activities ➔ Falls

### Post-traumatic stress disorder and psychological resilience

**Fig. 5:**
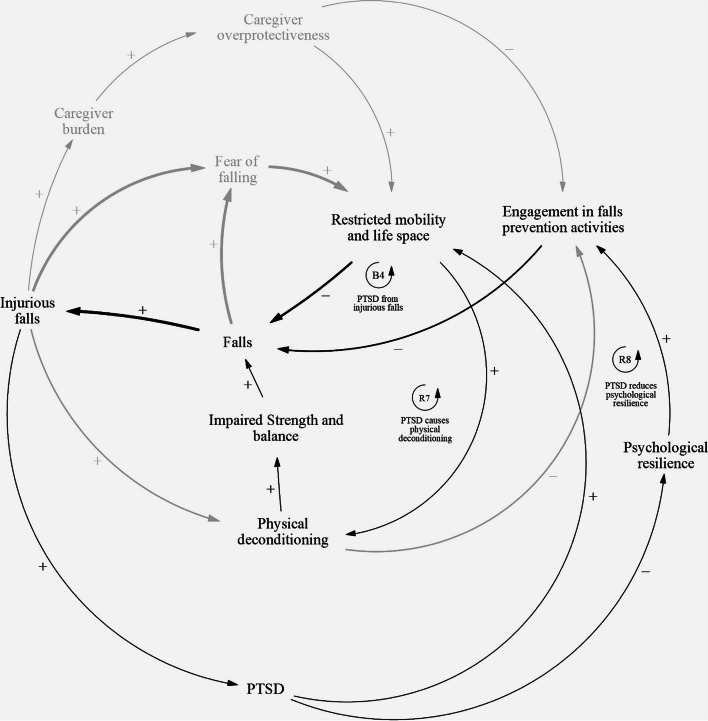
Causal pathways that involve post-traumatic stress disorder (PTSD)

The post-traumatic stress disorder (PTSD) sub-component of the causal loop diagram (Fig. 5) consists of three feedback loops: one balancing loop B4, and two reinforcing loops R7 and R8 (Table 5). Balancing loop B4 describes how PTSD may develop as a result of injurious falls. PTSD symptoms have been observed in fallers who suffer from an injurious fall. The traumatic experience of a fall, and the surrounding drama and stress associated with a serious fall may lead to PTSD development in fallers. Fallers may then restrict their mobility and life space, which would decrease the likelihood of another fall in the immediate future. Reinforcing loop R7 describes how PTSD causes physical deconditioning. The restriction of mobility and life space due to PTSD symptoms may lead to increased physical deconditioning, which would subsequently impair strength and balance in fallers and lead to an increased likelihood of falls in the long run. Reinforcing loop R8 describes how fallers who experience PTSD symptoms after an injurious fall have less psychological resilience. Fallers may experience negative moods and impaired cognition following a fall, and exhibit avoidance behaviours in response to stimuli associated with the fall. These symptoms may make it difficult for them to cope and fully recover from the fall, leading to reduced psychological resilience. As a result, they may become less active and motivated when participating in falls prevention programmes, which could reduce the effectiveness of falls prevention programmes that involve persistent, structured physical activities. Over time, this reduced engagement in falls prevention activities result in an increased likelihood of future falls.

**Table 5:** PTSD and psychological resilience feedback loops

Loop	Variables
B4	Falls ➔ Injurious Falls ➔ PTSD ➔ Restricted mobility and life space ➔ Falls
R7	Falls ➔ Injurious Falls ➔ PTSD ➔ Restricted mobility and life space ➔ Physical deconditioning ➔ Impaired strength and balance ➔ Falls
R8	Falls ➔ Injurious falls ➔ PTSD ➔ Psychological resilience ➔ Engagement in falls prevention activities ➔ Falls

### Predisposing factors for falls

**Fig. 6:**
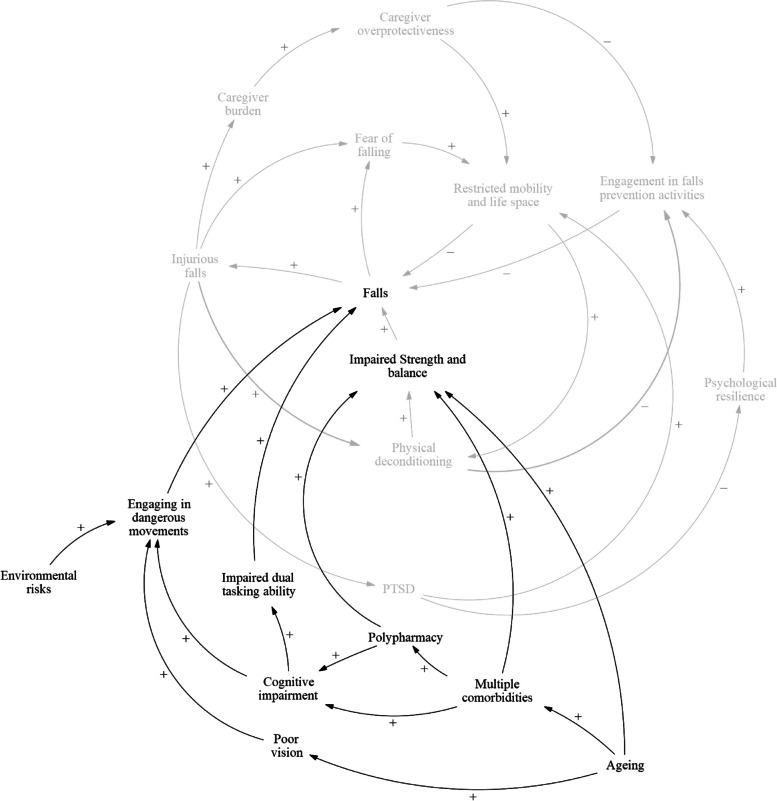
Causal loop diagram representing exogenous causes of falls that occur independently from a fall

During the GMB exercise, the participants proposed a series of risk factors that predispose older adults to falls that may not result from an index fall (Fig. 6). These factors can be classified broadly into two categories: physiological changes due to ageing and environmental factors. Firstly, physiological changes due to ageing typically include impaired strength and balance in fallers, which predisposes them to more falls. Ageing may result in the development of multiple comorbidities which may lead to a decline in physical and cognitive functions. Older adults with multiple comorbidities are often on several drug regimens for their chronic conditions. Polypharmacy and certain falls risk inducing medications may result in several side effects due to drug-drug interactions, which increases the risk of falls in older adults. Furthermore, cognitive impairment in older adults may result in impaired dual tasking abilities, predisposing them to falls. While performing daily activities, they may have to divide their attention between walking and other distractions. Cognitive impairment interferes with their ability to prioritize between different tasks and leads to a decline in motor performance when dual tasking. It also affects judgement and reflexes when they are moving about in a ‘dangerous environment’. The inability to perceive and adapt to dangers around them makes a cognitively impaired person more likely to fall. Impaired strength and balance in older adults also contribute to their unsteady movements and an increase their risk of falling. Older persons are also more likely to suffer from vision problems that affect their ability to navigate their environment safely.

Secondly, environmental factors, such as clutter, poor lighting, and slippery floors, were noted to contribute significantly to the risk of falling. Moreover, environmental factors could interact with age-related deficits such as cognitive or vision impairment that further increases the risk of falls.

### Key elements of effective falls intervention programmes

During the GBM exercise, the stakeholders identified the following elements as key to effective falls intervention programmes: physical training, environmental modifications, self-management, and goal setting (Table 6). Physical training for falls prevention focuses on building strength, balance, and endurance. The stakeholders noted that the physical training must be intense, consistent, and progressive for it to be effective. Training programmes that are tailored to suit individual needs and conditions are likely to achieve the best outcomes. Environmental modifications should be made in accordance with the needs of the individual, and specific to the neighbourhood they live in. Self-management by individuals is desirable to improve engagement in falls intervention programmes and promote self-efficacy. It can be facilitated using digital tools such as wearable devices to track physical activity or digital exercise diaries. Individual goal setting helps to motivate individuals to perform better in falls intervention programmes.

**Table 6:** Elements identified by the workgroup to be key to the effectiveness of falls intervention programmes

Falls intervention programmes should…
Focus on strength, balance and endurance
Have individualised environmental modifications
Have a component of self-management, possibly facilitated by technology
Have a component of goal setting as a driver for building motivation
Be acceptable to the community
Should be matched to a clearly defined population and their needs

Two contextual factors were noted to be important for the success of a falls prevention programme (Table 6). First, the programme should be acceptable to the community in which it is being held. Second, the programme should be tailored to the needs of a clearly defined population. Individualised physical exercise programmes with therapists may benefit those with multiple comorbidities and complex issues while community group exercises may benefit those who are not too disabled or cognitively impaired. Tailoring programs in this manner might not only improve their effectiveness, but also ensure that intensive resources are reserved for participants who are most likely to benefit.

### Mapping interventions to outcomes

**Fig. 7:**
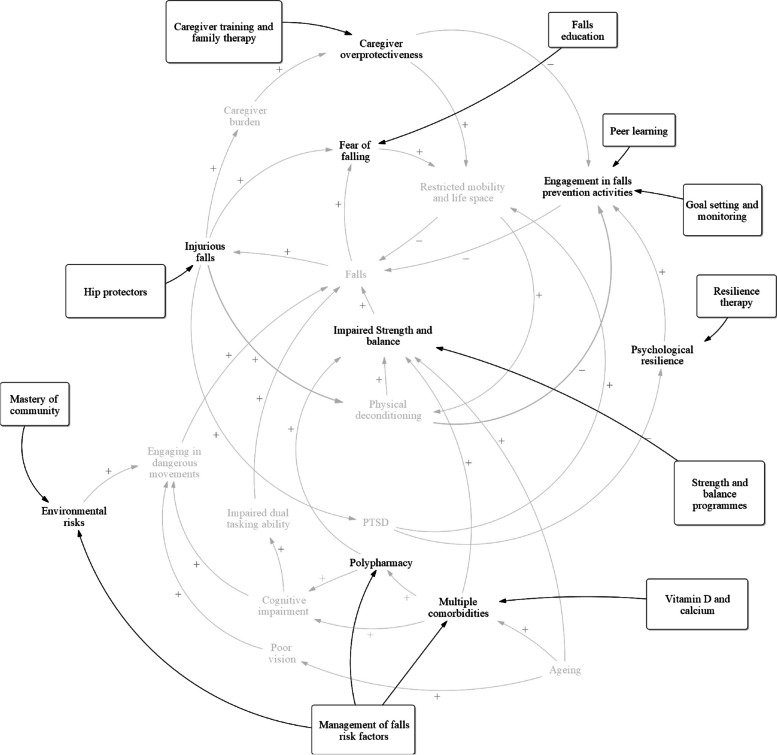
Causal loop diagram representing interventions and where they relate to on the causal loop diagram involving the causal pathways of falls

After constructing the CLD, we discussed specific falls intervention programmes in Singapore (Table 7) and how these are related to the variables identified in the causal loop diagrams (Fig. 7).

**Table 7:** Components of current falls intervention programmes conducted by the group of falls research stakeholders in Singapore

1) Management of falls risk factors
a. Balance and strength assessments
b. Polypharmacy related programmes
c. Footwear review
d. Home hazards assessment and modifications
e. Management of major comorbidities
1) Goals setting and monitoring
2) Strength and balance programme
3) Falls education
4) Peer learning
5) Mastery of community
6) Vitamin D and calcium
7) Hip protectors
8) Caregiver training and family therapy
9) Resilience therapy

All existing intervention programmes focus on improving strength and balance. They assess physical performance measures using multi-component tools such as the Short Physical Performance Battery. Impaired strength and balance play an important role in all four sub-components identified in the causal loop diagram and most directly influences the likelihood of future falls.

Falls education plays a role in the fear of falling sub-component of the CLD. Most extant programmes include some element of education to reduce the physical and psychological impact of falls. For instance, patients who are reasonably fit are taught how to roll in order to alleviate the risk of fractures. Some falls education programmes include a personal alarm system to alert others in the event of a fall; this can reduce prolonged distress after an unobserved fall as well as reduce delayed treatment of an injurious fall. The Stepping On programme has a cognitive behavioural component to reduce the fear of falling.

The use of hip protectors may minimise physical injuries resulting from falls in the injurious falls sub-component of the CLD, while peer learning, goal setting, and monitoring may play a role in improving engagement in falls prevention activities. None of the intervention programme conducted by members of the workgroup focused on medical treatment for improving bone density or on the use of devices such as hip protectors. However, existing falls intervention programmes indirectly help prevent Vitamin D deficiency by bringing individuals on walks during daytime. Peer learning facilitated by program leaders seeks to encourage engagement and improve self-efficacy in the Stepping On programme. Group leaders who encourage their peers to engage in falls prevention behaviour are identified and trained by healthcare professionals. Falls experts who are called in to conduct peer learning sessions also increase engagement as participants have few opportunities to encounter such experts outside of the programme. Goals setting and monitoring are a major part of the Stepping On Programme that encourages participants to track their progress throughout the fall intervention programme. This creates an active learning environment for the participants and encourages them to remain engaged in the community-based programme.

Furthermore, caregiver training and family therapy may reduce caregiver overprotectiveness in the caregiver overprotectiveness sub-component of the CLD. Caregivers are encouraged to understand falls intervention programmes and play a role in monitoring the participants’ exercise regimes. Caregiver education may help to tackle overprotectiveness of family members/ caregivers by helping them understand the importance of intense and persistent physical exercise in falls prevention.

In the PTSD and psychological resilience sub-component of the CLD, resilience therapy may improve faller’s ability to cope and adapt to life after a fall. However, none of the programmes explicitly aimed to improve psychological resilience that could encourage individuals to take charge of their own health, or to evaluate and manage people at risk of or manifesting PTSD after a fall.

Falls intervention programmes aim to mitigate predisposing factors for falls in older adults. For instance, programmes such as SAFE and physical therapy falls intervention clinics in the community, which are aimed at people who had experienced prior falls or otherwise identified as high risk for falls and injury, generally take a multicomponent approach. These programmes conduct a comprehensive screening of falls risk factors, followed by implementing tailored interventions that target these risk factors. Risk factors screened in falls intervention programmes include balance and strength, polypharmacy, major comorbidities, and home and environmental hazards. Participants are then risk stratified and assigned to intervention programmes that match their needs. Participants having vitamin D or calcium deficiency are identified by doctors and prescribed supplements. However, none of these intervention strategies have included screening for vitamin D and calcium deficiency in their programmes. The Stepping On programme includes a community navigation component where individuals are taught how to navigate their neighbourhood safely that helps them familiarise with their community.

## Discussion

In the presence of diverse clinical and research efforts to reduce falls in our rapidly ageing population, we described a GMB exercise that brought together different clinical and academic groups who are conducting falls research in Singapore. The exercise was aimed at clearly presenting the complex physical, emotional, and psychological sequelae that result from a fall and the interrelationships that exist between them. The exercise generated key insights on falls research, including (1) focusing on the psychological sequelae of falls as important outcomes in addition to looking at falls in and of itself, (2) identifying factors that may exacerbate the consequences of falls, such as overprotectiveness, psychological traits like resilience, or conditions like PTSD, and (3) adopting a multi-pronged approach to effectively limit the psychological consequences of falls.

### Focus on emotional and psychological sequelae of falls

In their efforts to identify key falls outcomes, the group suggested focusing on four key system outcomes in the falls intervention programmes: (1) falls, (2) injurious falls, (3) fear of falling, (4) restricted mobility and life space. The group learned that our definition of “falls related outcomes” should be broadened when measuring the success of multi-component falls intervention studies. Recent guidelines on falls assessment and intervention recommend the evaluation of the concern about falling in older adults, and a participatory approach to engaging older adults to achieve their goals [33]. These recommendations are in line with findings from our expert panel and indicate a broadening of goals for falls prevention in the community. While a reduction in falls and injurious falls are still important outcomes, addressing the emotional and psychological sequelae that results from these falls should also be part of the primary outcomes in falls intervention programs.

In every falls intervention study, falls are the primary outcome of interest [19, 21, 24]. Injurious falls have been increasingly identified as an outcome of importance to recognise the burden of falls on the healthcare system as falls are the main cause of trauma in older adults [7, 19].

However, fear of falling has only been recently recognised as an important psychological consequence of falls, which affects how fallers cope after a fall [9, 12]. Most interventional studies now include measures of falls efficacy to determine the effects of fear of falling, but few studies include the measure of fear of falling directly [20].

Restriction in mobility and life space has been recognised as an important measure of quality of life in a faller, but few interventional studies aim to improve mobility and life space of a faller directly [11, 34].

This GMB exercise is attempts to put together a broader definition of falls related outcomes and discuss the causal relationships between these key patient outcomes, for the first time. By doing so, falls research experts can better understand the role that each causal factor plays in contributing to the problem of falls in older adults, and the importance of measuring these key patient outcomes to eventually reduce the number of falls in the long run.

### Identifying factors that exacerbate the consequences of falls

The multifaceted relationship between falls risk and outcomes makes research and clinical practice complex. This GMB exercise described here is the first effort to visually represent the known and hypothesized causal relationships between these various facets of falls. The resulting CLD provided a framework for clear and structured communication between stakeholders. In particular, the GMB exercise allowed a group of falls research experts to identify areas where there may be insufficient evidence in the local context to support their ongoing hypotheses. Three such areas were identified: (1) the association between falls and restriction in mobility and life space; (2) the association between caregiver overprotectiveness and the effectiveness of falls intervention programmes; and (3) the association between PTSD or psychological resilience and falls prevention. These three novel areas of research in falls prevention have not been as well studied compared to other falls intervention components [33, 35].

In the CLD developed by the workgroup, restriction in mobility and life space is a component of the causal pathways of falls, fear of falling, and injurious falls leading to future falls. Furthermore, restriction in mobility and life space could possibly lead to fewer or more falls in the future, depending on whether and to what extent the faller experiences physical deconditioning as a result of the restriction. Across falls research literature, restriction in mobility and life space is well recognised as a detrimental outcome of a fall [11, 34]. However, qualitative studies on caregivers and fallers, consistently suggest that restrictions in mobility and life space of a faller develops, often with the intention or prevent falls in the future [36, 37]. Thus, future falls research could focus on how to change the perspectives of fallers and caregivers to prevent restriction in mobility and life space of a faller.

Besides the physical, emotional, and psychological effects of falls on the faller, falls also affect caregivers, family members, and friends who have witnessed them fall [36, 38, 39]. Overprotectiveness shown by family members or caregivers, by discouraging fallers from engaging in falls prevention activities altogether or with the intensity required to achieve a benefit, may reduce the effectiveness of falls prevention activities [40, 41]. Falls intervention research in Singapore should focus more on effectively addressing caregivers’ concerns so that they can support and encourage fallers to participate in falls prevention activities.

Recent evidence suggests that a significant proportion of individuals experiencing a fall, in particular an injurious fall, develop symptoms that fulfil DSM V criteria for PTSD [42, 43]. This finding suggests that PTSD treatments may be useful in alleviating the psychological trauma that results from a fall. Psychological resilience – the tendency to respond positively to life stressors – was judged to play a key role in the willingness of the faller to engage in falls prevention activities [44, 45]. More studies can be done in the local context to determine if psychological resilience is a modifiable risk factor to help fallers cope and adapt well after a fall.

### Adopting a multi-pronged approach in falls prevention

Once the causal relationships were established, the GMB exercise analysed how interventions can impact the dynamics of falls outcomes. This reinforced the general impression that the development of an effective falls prevention programme is inherently complex and difficult. Single intervention studies in Asia based on an exercise programme have been relatively successful in reducing falls-related outcomes [21, 33, 35, 46]. However, studies that simultaneously address multiple factors in the causal structure of falls, while routinely recommended, have not been as successful [46]. Recent consensus on community-based falls intervention programs recommends multi-component, multi-domain interventions to systematically screen for, and intervene to address different risk factors for falls [33]. This paper highlights how different components address the reinforcing dynamics of poor strength and balance in causing falls.

Three key insights relevant to multi-component interventions emerged from this GMB exercise. Firstly, literature on falls intervention research reinforces the notion that falls risk is often multifactorial, and that while physical injury is a major outcome, emotional and psychological sequalae have equally significant effects on an individual’s well-being after a fall [47–49]. The physical condition of the faller is relevant as individuals in poor physical condition are unable to engage meaningfully in falls prevention activities and therefore less likely to benefit from such programmes [19, 50].

Secondly, this GMB exercise has helped falls research experts to identify intermediate process measures that would potentially lead to positive falls intervention outcomes. Psychological resilience and alleviating caregiver overprotectiveness play important roles in determining the success of falls prevention programmes. As these measures are associated with better falls related outcomes [45, 51, 52], future falls research should focus more on them.

Thirdly, a systems approach to falls intervention helps us to identify specific causes of falls that are of importance to individual fallers to tailor multifactorial interventions [33]. While not all factors are of equal importance to every individual, this approach helps clinicians to identify how multi-component interventions can work synergistically to prevent falls. Multi-component falls intervention programmes need to be tailored for each faller to improve the effectiveness of the programme, according to the needs of the individual.

### Strength and limitations

The main strength of this GMB exercise is that it has brought together diverse and independent falls research groups in Singapore, to gain a common understanding of the causal structure of falls and develop a shared research agenda for future falls research and intervention programmes, for the first time.

However, one of the limitations of the exercise is that the causal structure is only a partial and likely simplified model of the real-world situations surrounding falls. We have only invited falls researchers with experience conducting falls intervention for community-dwelling older adults in Singapore, to represent their understanding of falls risk factors. Thus, this framework presents a preliminary framework which may be further validated by an independent group of research experts and clinicians, who can critically analyse its structure using the Categories of Legitimate Reservation approach. Once validated, it can be used as a framework that will guide future research and implementation [30]. Moreover, the workgroup conducted the exercise based on their understanding of the falls problem and did not systematically review the literature generally or for the Asian context. Thus, the validation effort would also serve to improve the CLD by adding or eliminating structures or redefining entities within the diagram.

This paper also describes the understanding of current falls research in Singapore from the perspective of clinicians and falls researchers. The perspective of fallers is important in fully understanding the conceptual dynamics in the causes of falls that was not the focus of this study. Further studies will be conducted to capture this perspective using qualitative methods to interrogate their lived experiences of falls in the community.

### Implications

The work described here has several implications. First, it illustrates the use of GMB as a tool to promote shared understanding of complex health issues, an approach that can be adopted by a diverse group of stakeholders, including policy experts, clinicians, allied health professionals, and academic researchers [53, 54]. Second, it can create a shared research agenda for improving efforts to reduce the burden associated with falls. The causal loop structure helped the group to identify factors that may play an important role in determining the physical, social, and psychological sequelae of falls, and therefore needs to be investigators further. The current exercise led to the identification of three such factors—family overprotectiveness, psychological resilience, and PTSD. Third, the insights that emerged from this exercise could be translated into policy decisions to extend the impact of falls implementation research to a larger group of older adults. For example, the exercise highlighted the importance of adopting more tailored approaches to address certain aspects of the falls problem that are relevant to specific patient groups and environments.

## Conclusion

In this study, Group Model Building was used as a tool to bring together falls research experts in Singapore to discuss on their shared mental models on the causal structure of falls among older adults in the community. The work group then examined specific areas in the causal structure of falls that were tackled by previous falls intervention programmes and identified areas for further falls intervention research whose findings could promote more informed clinical and policy decision making.

### Supplementary Information

**Additional file 1.**

## Data Availability

The models used and/or analysed during the current study available from the corresponding author on reasonable request.

## Abbreviations

GMB: Group Model Building
CLD: Causal Loop Diagram
PTSD: Post-traumatic stress disorder
DSM-V: Diagnostic and Statistical Manual of Mental Disorders, Fifth Edition
