# Supplementary material for: Group Model Building on causes and interventions for falls in Singapore: insights from a systems thinking approach
Source: BMC Geriatr. 2023 Sep 22;23:586. doi: 10.1186/s12877-023-04294-2 (PMC10517526; doi:10.1186/s12877-023-04294-2)

**Appendix 1: Implementation Research on Falls Script**

**#1: Hopes and Fears for Falls Implementation Research Script**

| ***Description*** | To discuss a range of hopes and fears about the direction of falls implementation research and its potential impact. |
| --- | --- |
| ***Context*** | After the presentation of the 4 teams and their falls intervention programmes |
| ***Primary nature of the group*** | Individual |
| ***Time*** | Prep time: none  Time duration: 40 minutes  Follow-up time: none |
| ***Materials*** | None |
| ***Inputs*** | None |
| ***Outputs from this script*** | List of hopes and fears about the direction of falls implementation research and its potential impact. |
| ***Roles*** | Facilitator, recorders, observers |
| ***People in the room*** | All participants |
| ***Steps*** | 1. Each participant was to voice out one hope or one fear in a round-robin format. 2. The facilitator will ask questions to get the participants to elaborate further on what they have voiced out. |
| ***Evaluation Criteria*** | Participants were able to understand the overall exercise and were engaged throughout the process. |
| ***Reference*** | Andersen, D. F., & Richardson, G. P. (1997). Scripts for group model building. *System Dynamics Review, 13*(2), 107-129.  Luna-Reyes, L. F., Martinez-Moyano, I. J., Pardo, T. A., Cresswell, A. M., Andersen, D. F., & Richardson, G. P. (2006). Anatomy of a group model-building intervention: Building dynamic theory from case study research. System Dynamics Review, 22(4), 291-320. |

**#2: Discussion of Desirable Outcomes**

| ***Description*** | The process to reflect on key outcomes that are most important in falls prevention among older adults. Participants reflected on whether these outcomes are desirable in itself and discussed which of these are most important and worth paying for. |
| --- | --- |
| ***Context*** | After the hopes and fears exercise. |
| ***Primary nature of the group*** | Group |
| ***Time*** | Prep time: none  Time duration: 120 minutes  Follow-up time: none |
| ***Materials*** | - Two different colours of post-it sheets (4 x 6 in) - Felt pens - Sticky sheets to stick a post-it on |
| ***Inputs*** | None |
| ***Outputs from this script*** | List of outcomes for falls intervention research. Causal map of falls risk factors that lead to falls. |
| ***Roles*** | Facilitator, recorders, observers |
| ***People in the room*** | All participants |
| ***Steps*** | 1. Each group (3 groups) is given multiple post-it sheets (4 x 6 in) 2. Facilitator asks participants the guiding question— **“What are some of the outcomes of falls research that you feel are most important? “** 3. Each participants brainstorm and list outcomes on a post-it (one outcome per post-it) (max. 20 minutes). 4. In a round-robin fashion, each person per group reads out one outcome at a time; the facilitator collects the post-it (ask clarifying questions) and sticks it on the white wall. 5. Facilitator clusters the post-it into themes. 6. The facilitator probes participants about the nature of the causal relationships and their polarity, while drawing the connections on the white sheet. Participants are also encouraged to identify feedback loops. 7. The recorder for the session writes down the list of all outcomes and takes pictures. |
| ***Evaluation Criteria*** | Participants were able to understand the overall exercise and were engaged throughout the process. |
| ***Reference*** | Andersen, D. F., & Richardson, G. P. (1997). Scripts for group model building. *System Dynamics Review, 13*(2), 107-129.  Luna-Reyes, L. F., Martinez-Moyano, I. J., Pardo, T. A., Cresswell, A. M., Andersen, D. F., & Richardson, G. P. (2006). Anatomy of a group model-building intervention: Building dynamic theory from case study research. System Dynamics Review, 22(4), 291-320. |

**#3: Current Programmes and Interventions Script**

| ***Description*** | The process to highlight key intervention elements of effective falls intervention programmes |
| --- | --- |
| ***Context*** | After the Elicitation of Desirable Outcomes exercise |
| ***Primary nature of the group*** | All participants |
| ***Time*** | Prep time: 15 minutes  Time duration: 120 minutes  Follow-up time: none |
| ***Materials*** | - White Post-it sheet (big size) - Thick writing markers |
| ***Inputs*** | The list of outcomes and the conceptual model |
| ***Outputs from this script*** | A list of variables that the group considers as key intervention elements. |
| ***Roles*** | Facilitator, recorder, observers |
| ***People in the room*** | All participants |
| ***Steps*** | 1. Facilitator present the causal diagram on the factors that result in desirable outcomes in falls intervention based on input from participants from the second exercise) on a large white sticky sheet pasted on a blank wall. 2. Participants are encouraged to suggest key intervention elements of falls intervention programs, and write them on post-it notes. 3. The facilitators will ask clarifying questions about each intervention element. 4. The facilitators will then summarise the current efforts and gaps in falls research in Singapore. 5. The recorder for the session takes notes of the intervention elements. |
| ***Evaluation Criteria*** | Participants were able to understand the overall exercise and were engaged throughout the process. |
| ***Reference*** | Andersen, D. F., & Richardson, G. P. (1997). Scripts for group model building. *System Dynamics Review, 13*(2), 107-129.  Luna-Reyes, L. F., Martinez-Moyano, I. J., Pardo, T. A., Cresswell, A. M., Andersen, D. F., & Richardson, G. P. (2006). Anatomy of a group model-building intervention: Building dynamic theory from case study research. System Dynamics Review, 22(4), 291-320. |

**#4: Mapping of Current Interventions to Falls Outcomes Script**

| ***Description*** | The process to map the current interventions to the falls outcomes. |
| --- | --- |
| ***Context*** | After the presentation of the functional loss and recovery model. |
| ***Primary nature of the group*** | Groups |
| ***Time*** | Prep time: none  Time duration: 60 minutes  Follow-up time: none |
| ***Materials*** | - White Post-it sheet (big size) - Thick writing markers - Sticky boards to stick sheets |
| ***Inputs*** | A list of variables that the group considers as key intervention elements. |
| ***Outputs from this script*** | A causal map of falls intervention elements to the causal diagram on falls. |
| ***Roles*** | Facilitator, recorder, observers |
| ***People in the room*** | All participants |
| ***Steps*** | 1. Facilitator present the causal diagram on the factors that result in desirable outcomes in falls intervention based on input from participants from the second exercise) on a large white sticky sheet pasted on a blank wall, and the list of key intervention elements highlighted in third exercise. 2. Participants are encouraged to suggest which falls risk factors are addressed by the key intervention elements of falls intervention programs. 3. The facilitators will build the wall by taking the sticky notes from the participants, ask clarifying questions about the relationships the participants have suggested and place them on the causal map of falls. 4. The facilitators will then draw arrows to suggest areas addressed by interventions in Singapore. 5. The facilitator summarise the current efforts and gaps in falls research in Singapore. 6. The recorder for the session take notes of the complete causal map. |
| ***Evaluation Criteria*** | Participants were able to understand the overall exercise and were engaged throughout the process. |
| ***Reference*** | Andersen, D. F., & Richardson, G. P. (1997). Scripts for group model building. *System Dynamics Review, 13*(2), 107-129.  Luna-Reyes, L. F., Martinez-Moyano, I. J., Pardo, T. A., Cresswell, A. M., Andersen, D. F., & Richardson, G. P. (2006). Anatomy of a group model-building intervention: Building dynamic theory from case study research. System Dynamics Review, 22(4), 291-320. |

**Appendix 2: Symbols and Notations used in Causal Loop Diagrams**


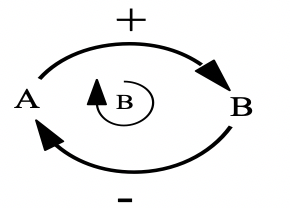

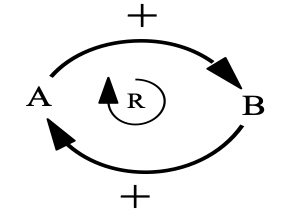

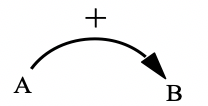

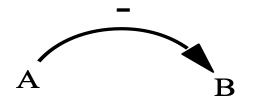

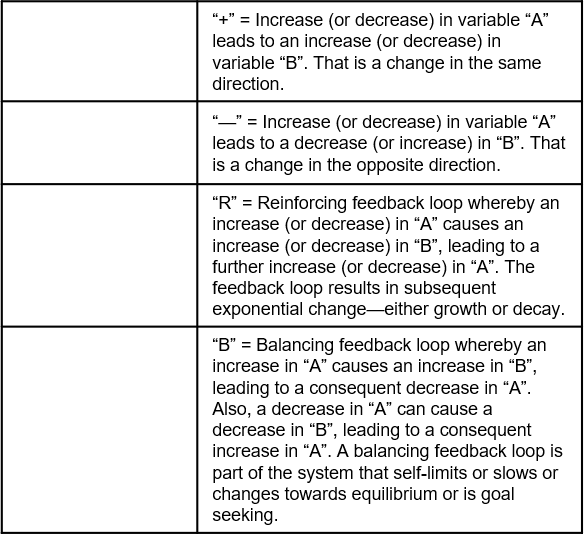

Supplement: Supplementary file 1 — Additional file 1. [file 12877_2023_4294_MOESM1_ESM.docx]
